# Supplementary material for: Importance of active layer freeze-thaw cycles on the riverine dissolved carbon export on the Qinghai-Tibet Plateau permafrost region
Source: PeerJ. 2019 Jun 17;7:e7146. doi: 10.7717/peerj.7146 (PMC6585901; doi:10.7717/peerj.7146)
Supplement: Supplemental Information 2 — The supplementary figures contain information about photos of the study area (Figure S1), thawed depth variations in the thawing period (Figure S2), frozen depth variations in the freezing period (Figure S3), soil moisture and temperature variations in 2014 and 2016 (Figure S4), cation compositions of Fenghuoshan catchment (Figure S5), time series of measured DIC and DOC of the sub-catchments (Figure S6), and DIC:DOC ratio in 2014 and 2016 frozen-free season (Figure S7). [file peerj-07-7146-s002.pdf]

Supplementary Information

Importance of active layer freeze-thaw cycles on the riverine dissolved carbon export on the Qinghai-Tibet Plateau permafrost region

Chunlin Song[1, 2], Genxu Wang*[1], Tianxu Mao[3], Xiaopeng Chen[1, 2], Kewei Huang[1, 2], Xiangyang Sun[1], Zhaoyong Hu[1, 2]

[1]Institute of Mountain Hazards and Environment, Chinese Academy of Sciences, Chengdu 610041, People's Republic of China

[2]University of Chinese Academy of Sciences, Beijing 100049, People's Republic of China

[3]College of Forestry, Guizhou University, Guiyang 550025, People's Republic of China

Corresponding author: Genxu Wang (Email: wanggx@imde.ac.cn, phone: +86 28 85233420, fax: +86 28 85222258).

Contents of this file

Figures S1 to S7

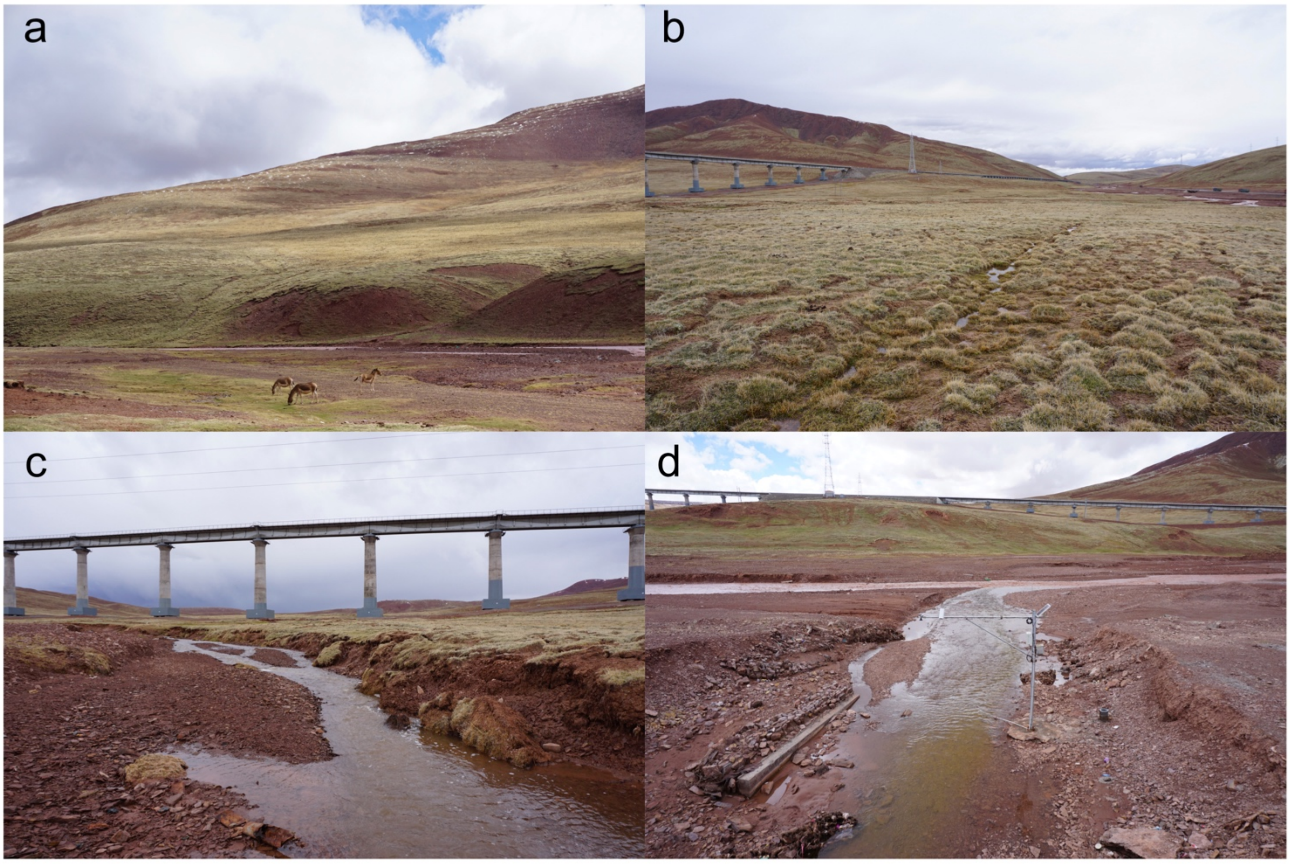

Figure S1. Photographs of the study watershed hillslope landscape (a), alpine meadow vegetation (b), Catchment 5 (c) and Catchment 3 (d). Photo credit: Chunlin Song.

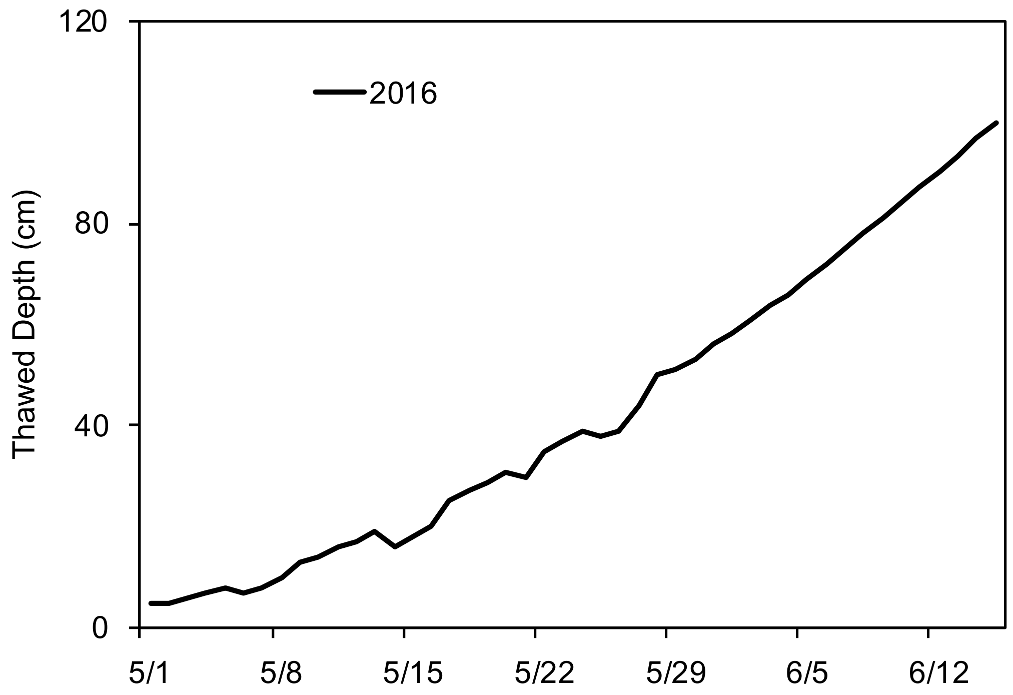

Figure S2. The thawed depth of active layer in 2016 thawing period.

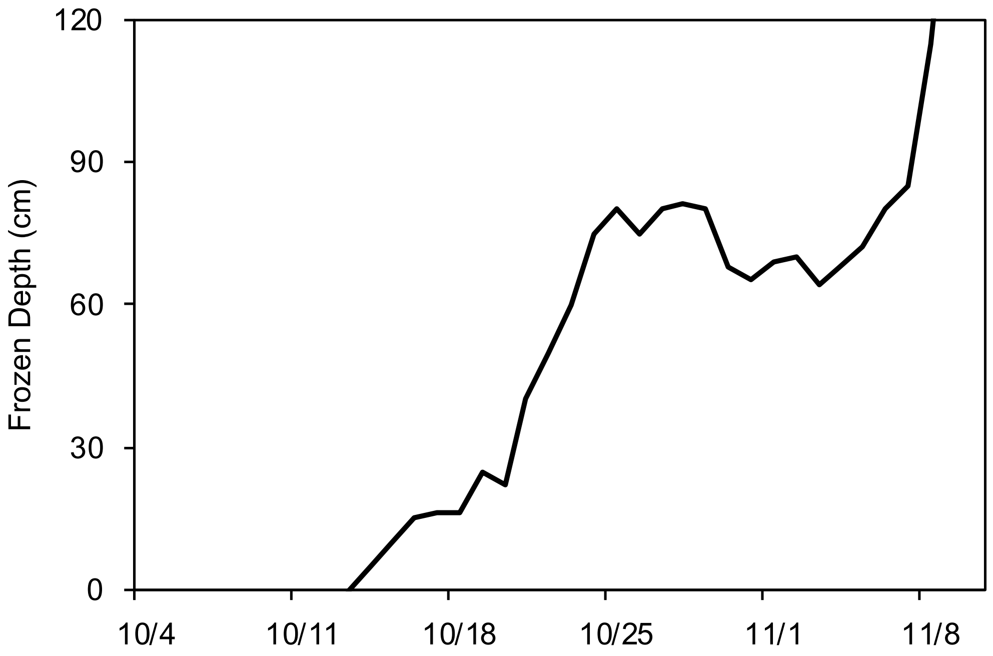

Figure S3. The frozen depth of active layer in 2016 freezing period.

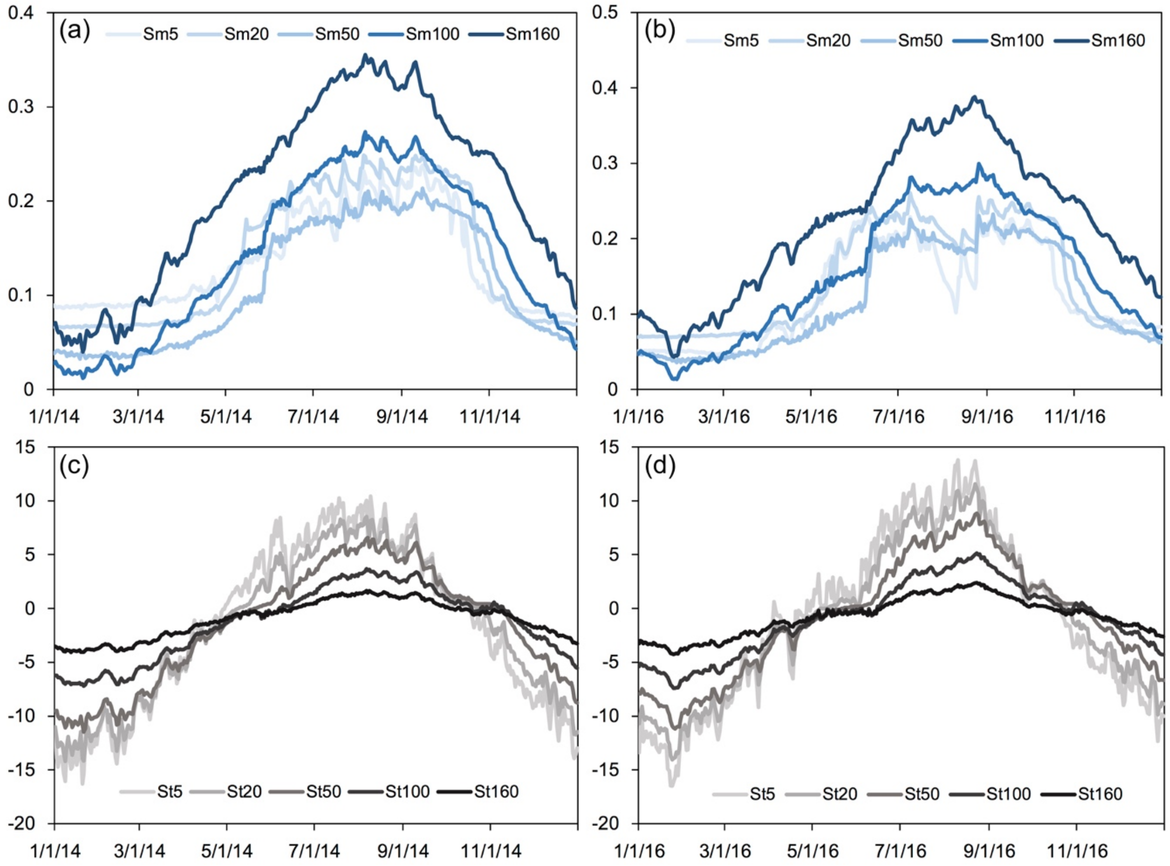

Figure S4. The daily mean soil moisture and temperature at different depths of our study site in 2014 and 2016. Sm5, Sm20, Sm50, Sm100, and Sm160 are soil moisture at depths of 5, 20, 50, 100, and 160 cm, respectively; St5, St20, St50, St100, and St160 are soil temperature in °C at depths of 5, 20, 50, 100, and 160 cm, respectively.

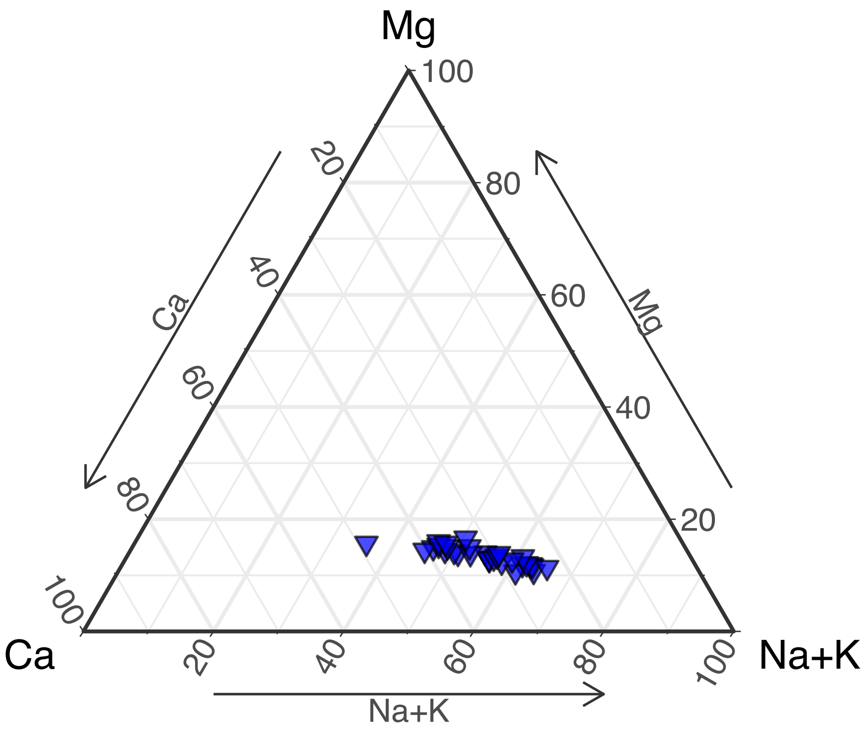

Figure S5. Ternary diagram showing the cation composition of the Fenghuoshan watershed.

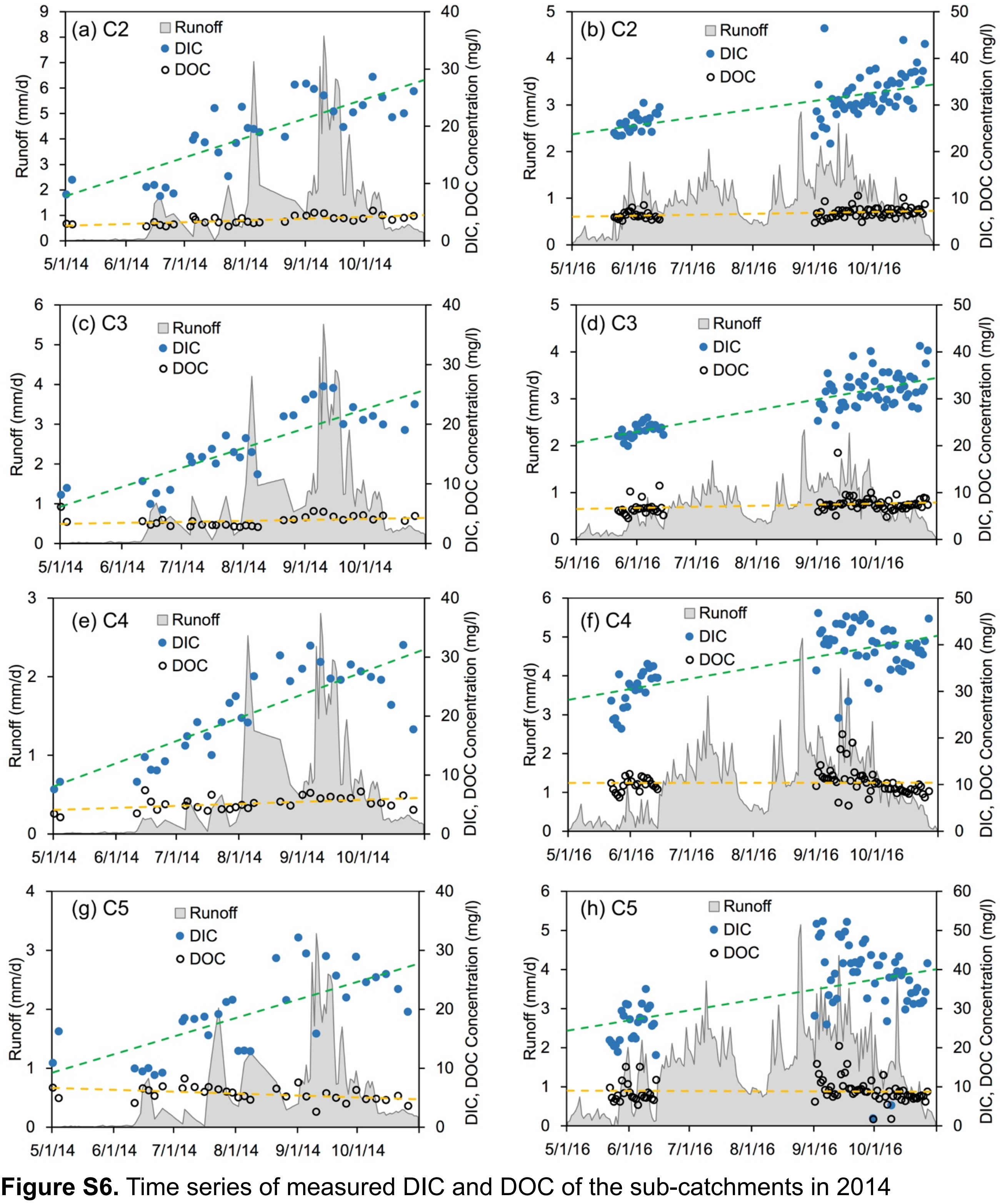

Figure S6. Time series of measured DIC and DOC of the sub-catchments in 2014 and 2016. In the figures C2, C3, C4 and C5 means Catchment 2, Catchment 3, Catchment 4, and Catchment 5, respectively. Date format: month/date/year.

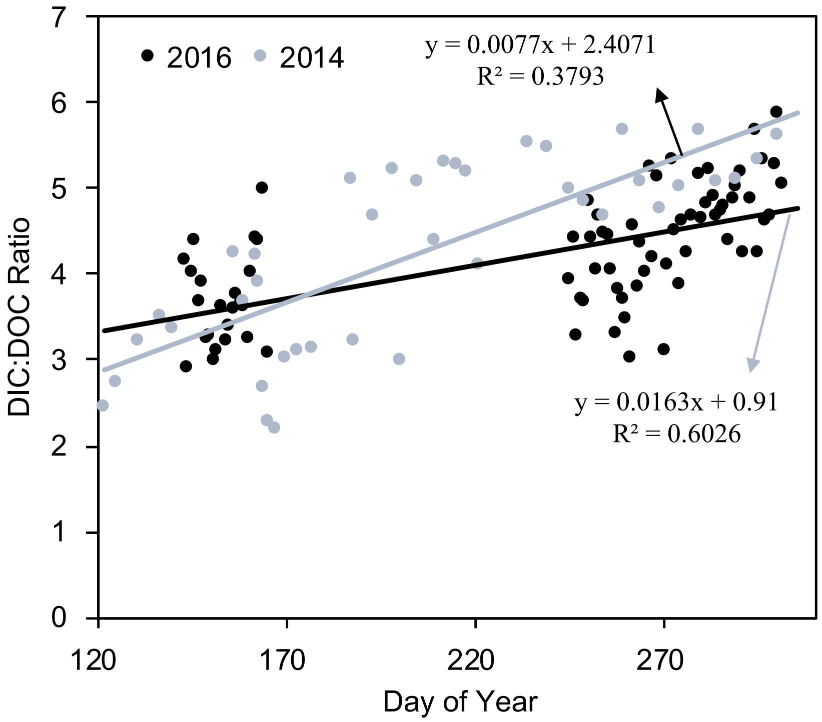

Figure S7. The ratio of DIC:DOC concentration throughout the study period in 2014 and 2016.
